# Regenerative Repair of Damaged Meniscus with Autologous Adipose Tissue-Derived Stem Cells

**DOI:** 10.1155/2014/436029

**Published:** 2014-01-30

**Authors:** Jaewoo Pak, Jung Hun Lee, Sang Hee Lee

**Affiliations:** ^1^Stems Medical Clinic, 32-3 Chungdam-dong, Gangnam-gu, Seoul 135-950, Republic of Korea; ^2^National Leading Research Laboratory, Department of Biological Sciences, Myongji University, 116 Myongjiro, Gyeonggido, Yongin 449-728, Republic of Korea

## Abstract

Mesenchymal stem cells (MSCs) are defined as pluripotent cells found in numerous human tissues, including bone marrow and adipose tissue. Such MSCs, isolated from bone marrow and adipose tissue, have been shown to differentiate into bone and cartilage, along with other types of tissues. Therefore, MSCs represent a promising new therapy in regenerative medicine. The initial treatment of meniscus tear of the knee is managed conservatively with nonsteroidal anti-inflammatory drugs and physical therapy. When such conservative treatment fails, an arthroscopic resection of the meniscus is necessary. However, the major drawback of the meniscectomy is an early onset of osteoarthritis. Therefore, an effective and noninvasive treatment for patients with continuous knee pain due to damaged meniscus has been sought. Here, we present a review, highlighting the possible regenerative mechanisms of damaged meniscus with MSCs (especially adipose tissue-derived stem cells (ASCs)), along with a case of successful repair of torn meniscus with significant reduction of knee pain by percutaneous injection of autologous ASCs into an adult human knee.

## 1. Introduction

The menisci are two semilunar, fibrocartilaginous disks located between the medial and lateral articular surfaces of the femur and tibia in each knee. Their key functions are (i) the transfer of weight, (ii) absorption of shock during dynamic movements of the knee, and (iii) protecting the cartilage in the joint [1]. Therefore, damage in the meniscus can cause continuous knee pain. The meniscal damage can be diagnosed usually by physical exam and confirmed by magnetic resonance imaging (MRI) scans [2]. With diagnosis, the knee pain due to meniscus tear is initially treated conservatively [3, 4]. If the initial conservative treatment fails, a meniscectomy is normally performed. However, the current treatment with meniscectomy, even with partial one, is associated with early onset of osteoarthritis of the knees [5–8]. Thus, the lack of noninvasive cure for the menisci damage presents a major therapeutic challenge. However, recent studies have shown possible articular cartilage regeneration using mesenchymal stem cells (MSCs) in human patients with osteoarthritis and chondromalacia patellae [9–11]. The same MSCs have also been shown to regenerate bone-like tissue in osteonecrosis patients [12]. However, there has been no report of fibrous cartilage restoration in a patient with meniscus tear. Further, from June 2009, the Korean Food and Drug Administration has allowed medical uses of nonexpanded stem cells processed in a medical facility [13]. Here, we describe the possible regenerative mechanisms of damaged meniscus with MSCs (especially adipose tissue-derived stem cells (ASCs: one kind of MSCs)) and the first successful approach to reduce the knee pain by percutaneously injected autologous ASCs along with platelet-rich plasma (PRP), hyaluronic acid and CaCl_2_, an ASC mixture.

## 2. Regenerative Mechanisms of Damaged Meniscus

### 2.1. Direct Differentiation of the Stem Cells (MSCs)

MSCs were first identified and isolated from bone marrow (BM) in the late 1960s [14]. Subsequent studies demonstrated that these BM-derived MSCs possess self-renewal capacity and capability to form other cell types, such as adipocytes, osteoblasts, and chondrocytes [15]. Later, the International Society for Cellular Therapy (ISCT) established minimal criteria for defining MSCs based on characteristics found in the BM-derived MSCs.

According to ISCT, the criteria for identification of MSCs are as follows: (i) MSCs must adhere to plastic under standard culture conditions; (ii) MSCs must express the surface molecules CD105, CD73, and CD90 and lack expression of CD45, CD34, CD14 or CD11b, CD79*α* or CD19, and HLA-DR; and (iii) MSCs must have the capability to form osteocytes, adipocytes, and chondrocytes [16]. Since then, numerous studies have identified and isolated MSCs in almost all animal tissues, including human [17–20]. MSCs are, thus, defined to be pluripotent cells found in numerous human tissues, including BM and adipose tissue. Such MSCs isolated from BM and adipose tissue have been shown to differentiate into bone, cartilage, and adipose tissue [21, 22].

Human ASCs were first discovered and identified in the early 2000s in the form of stromal vascular fraction (SVF) and shown to possess self-renewal capacity and the capability to differentiate into adipocytes, osteoblasts, and chondrocytes [21–24]. In addition, ASCs were more readily available and were relatively easier to be isolated than BM.

Currently, ASCs have been shown to differentiate toward cells that are mesodermal and nonmesodermal in origin. In 2005, Brzoska et al. reported *in vitro* differentiation of ASCs into epithelial-like cells [25]. Subsequently, ASCs were shown to undergo differentiation into cells resembling corneal keratocytes, retinal pigment epithelial-like cells, dental bud-like structures, and renal tubular epithelium [26–29]. The capability of ASCs to differentiate into endodermal cell types has also been reported. ASCs have been shown to undergo hepatic differentiation and they could be able to engraft into the liver and reconstitute some hepatocyte functions [30–34]. ASCs have also been shown to form pancreatic islet-like cells, excreting insulin and glucagon [35–37].

In case of meniscus repair, a few studies showing the direct differentiation of MSCs into meniscal cells were reported in animal models. Mizuno et al. reported that synovium-derived MSCs were able to attach to the meniscal defect of a rat and differentiate into cartilage cells [38, 39]. Another study showed that synovium-derived MSCs injected into rat knees adhered to the massive meniscal defects and differentiated into meniscal cells directly [40]. Zellner et al. showed that precultured BM-derived MSCs resulted in the regeneration of avascular meniscal tears with differentiated stable meniscus-like tissue in a rabbit model [41]. There was only one report showing that transplanted ASCs formed meniscal fibrocartilage in the healed zones of a rabbit [42]. These results indicated that transplanted MSCs could differentiate into meniscal cells and promote meniscal regeneration.

Potential mechanisms for regeneration and repair of damaged meniscus by MSCs (especially ASCs) can include the following: (i) transplanted cells differentiate into de novo tissue [42]; (ii) stem cells within the transplant replenish progenitor cells in the host [43]; and/or (iii) transplanted cells may fuse with host cells (in a process known as cell fusion) [43].

### 2.2. Trophic and Paracrine Effects of MSCs (Especially ASCs) on the Existing Meniscal Cartilage

Although the potential for ASCs to differentiate into various tissues of the body seems to be limitless, the ability of ASCs to promote tissue regeneration and repair may also depend on their paracrine effects. ASCs have the capability to secrete various growth factors that can modulate host tissue environment [44]. Such growth factors have been shown to have paracrine effects via neovascularization and immunosuppression. Neovascularization is a mechanism through which ASCs promote host tissue repair [45]. ASCs secrete vascular endothelial growth factor (VEGF) and hepatocyte growth factor (HGF) for neovascularization. These growth factors have been implicated in the ability of ASCs to repair scarred myocardium [45, 46]. Human ASCs have also been shown to promote revascularization of ischemic mouse hind limbs through HGF secretion, possibly via neovascularization [47, 48]. Such pro-angiogenic and paracrine effects may also contribute to the ability of ASCs to facilitate recovery from cerebral vascular injuries and account for ASC utility in the treatment of erectile dysfunction [49–51].

When immunity has been oversurmounted, lymphocytes and mononuclear cells can damage the host tissue. ASCs can promote tissue repair and regeneration through immune-suppressing lymphocytes and peripheral blood mononuclear cells, thus preventing overinflammation [52–54]. Some reports have shown that ASC-conditioned medium attenuates lymphocyte proliferation suggesting that ASCs secrete immunosuppressive factors [55, 56]. Proposed candidates for immunosuppressive effects of ASCs include prostaglandin E2, leukemia inhibitor factor, and kynurenine, a product of tryptophan metabolism [55–58]. When ASCs were cocultured with lymphocytes, ASCs increased their secretion of each of these molecules [55–58]. In addition, when ASCs were inhibited from producing these agents, the suppressive effects on lymphocyte proliferation by ASCs were not provided [55–58].

In case of meniscal repair, implanted BM-derived MSCs can produce factors that inhibit scarring (fibrosis) and apoptosis, promote angiogenesis, and stimulate host progenitors to divide and differentiate into functional regenerative units [59]. Zellner et al. reported that BM-derived MSCs can promote meniscal healing in a rabbit model by synthesizing and secreting a broad spectrum of growth factors and cytokines, which can initiate a cascade of repair mechanisms and attract repair cells from the surrounding tissue [60]. There was no study showing that transplanted ASCs stimulated meniscal regeneration and repair.

Although it is not clear, at this time, if paracrine effects via neovascularization and immunosuppression are involved in regeneration and repair of meniscus, it is very possible that one of the many growth factors excreted by ASCs can have a profound trophic, paracrine effect on the surrounding intact meniscus.

### 2.3. Effects of Growth Factors Contained in PRP

Understanding platelet physiology has led to the concept of utilizing platelet growth factors in natural regenerative therapies. A simple centrifugation system can isolate and concentrate platelets from an autologous blood sample, producing platelet-rich plasma (PRP). PRP contains a high concentration of autologous growth factors. With activation, platelet degranulation is induced and a concentrated pool of autologous growth factors can be released to injured site to augment natural regenerative pathways [61, 62]. This process of degranulation initiates cellular proliferation and tissue repair responses. Some of the growth factors that are released during degranulation process are platelet-derived growth factor (PDGF), transforming growth factor-*β*, vascular endothelial growth factor (VEGF), and epithelial growth factor (EGF) [61, 62].

Clinical applications of PRP have so far demonstrated encouraging therapeutic results. Mishra and Pavelko reported greater than 90% reduction in elbow pain with PRP injections of chronic elbow tendinosis [63]. Aspenberg and Virchenko verified by histology that a single PRP injection into a transected rat Achilles tendon had a greater maturation of tendon cells than control [64]. Anitua et al. hypothesized that the released growth factors from PRP have a chemotactic and mitogenic effect on MSCs [65]. These authors have also hypothesized that growth factors from PRP may also promote angiogenesis, stimulation of cellular protein synthesis, tissue remodeling, and formation of new extracellular matrix (ECM). In 2006, Eppley et al. reported that PRP stimulated endothelial cells for possible formation and proliferation of new capillary vessels [62]. Moreover, in 2009, Hu et al. observed expression of VEGF and PDGF in bone marrow induced by PRP and concluded that PRP can be a potential contributor in initiating angiogenesis [66]. Further, PRP has been shown to stimulate stem cells to proliferate as well as differentiate [67, 68].

Growth factors in PRP have been shown to enhance the healing properties of the inner avascular part of rabbit meniscal cells and to enhance the biological activities of the meniscal cells for meniscal tissue regeneration [69, 70].

Potential mechanisms for possible regeneration of the damaged meniscus by PRP can include the following: (i) platelet growth factors can be released to damaged meniscus to augment natural regenerative pathways; (ii) the released growth factors from PRP have a chemotactic and mitogenic effect on MSCs; (iii) PRP can be a potential contributor in initiating angiogenesis; and/or (iv) PRP has been shown to stimulate stem cells to proliferate as well as differentiate.

### 2.4. Extracellular Matrix Production by Chondrocytes Stimulated by Low Physiologic Doses of Dexamethasone

In addition to PRP growth factors, various other elements can affect MSC differentiation in their natural environment [71–75]. One of the elements of note is the physiologic doses of dexamethasone. Several authors have shown that, *in vitro*, nanodoses of dexamethasone influence MSCs to differentiate toward chondrogenic [76–78] or osteogenic [79, 80] lineage. Although, *in vivo*, physiologic doses of dexamethasone are present for MSCs to differentiate toward chondrogenic or osteogenic lineage, additional supplement of physiologic doses of dexamethasone with transplanted MSCs would ensure differentiation of MSCs to chondrocytes or osteogenic cells. Although it is not clear, it is very possible that the MSCs' differentiation induced by low physiologic doses of dexamethasone can affect regenerative repair of damaged meniscus.

## 3. Clinical Application of ASCs to Repair Damaged Meniscus

According to Korean law (Rules and Regulations of the Korean Food and Drug Administration), this study does not need approval by ethics and science committees [13]. Further, this clinical study was in compliance with the Declaration of Helsinki and regulation guidelines of the Korean Food and Drug Administration. An informed consent was obtained from the patient.

### 3.1. Materials and Methods

The inclusion criteria, exclusion criteria, and outcome endpoints are listed in Tables 1, 2, and 3.

For pain score, functional rating index, visual analog scale (VAS), physical therapy (PT), and range of motion (ROM) were determined as previously described [81, 82].

The patient was restricted from taking steroids, aspirin, nonsteroidal anti-inflammatory drugs (NSAIDs), and Asian herbal medications for one week prior to the procedure.

In the operating room, approximately 40 mL of packed adipose tissue was obtained by liposuction of the subcutaneous layer of the lower abdominal area using sterile techniques [11]. The stromal vascular fraction (SVF) containing ASCs was separated from the lipoaspirates by a fat stem cell isolator (SCELDIS, ED Co., Ltd., Republic of Korea) after treatment with collagenase.

ASCs were extracted through the use of digestive enzymes (0.07% type 1 collagenase; Adilase, Worthington, Lakewood, NJ, USA) and centrifugation (500 g) [11, 12, 24]. The total volume of the solution containing ASCs was 8.5 mL. While preparing the ASCs, 30 mL autologous blood were drawn along with 2.5 mL anticoagulant citrate dextrose solution (0.8% citric acid, 0.22% sodium citrate, and 0.223% dextrose; Baxter Healthcare Corp., Marion, NC, USA). After centrifugation (100 g, then 1000 g), 4.4 mL of platelet-rich plasma (PRP) along with the Buffy coat was obtained. Hyaluronic acid (0.5% (w/v), 2 mL; Huons, Chungbuk, Korea) was added as a scaffold to this mixture, and 3% (w/v) CaCl_2_ (0.1 mL; Choongwae Pharmaceutical Co., Gyeonggido, Korea) was added to activate PRP. These ASCs along with PRP, hyaluronic acid, and CaCl_2_ stand for the ASC mixture.

After the left knee was cleaned with 5% povidone-iodine (Choongwae Pharmaceutical Co., Seoul, Korea) and draped in a sterile fashion, the injection site was anesthetized with 2% lidocaine (Daehan Pharmaceutical Co., Gyeonggido, Korea). On the day of liposuction, the ASC mixture (15 mL) was injected into the medial Tibiofemoral joint and into the medial inferior retropatellar joint on the day of liposuction with a 20-gauge, 1 1/2-inch needle under ultrasonic guidance. On the third and seventh day after the initial injection, another dose of PRP with CaCl_2_ and hyaluronic acid (1 mL) was injected in the same fashion as the first day. On the fourteenth day after the initial injection, a low-dose (254.8 nmol/L) dexamethasone (Huons, Chungbuk, Korea) was added to PRP with CaCl_2_. On day 28, the last dose of PRP with CaCl_2_ was injected.

The patient was followed up with telephone questionnaires every six months at 6, 12, and 18 months. Each time, the patient was asked the following questions. (i) Was the symptom improvement persistent? (ii) Did you experience any complications (e.g., infection, illness) you believe may be due to the procedure? If yes or maybe, please explain. (iii) Have you been diagnosed with any form of cancer since the procedure? If yes, please explain.

### 3.2. Result

**Figure 1:**
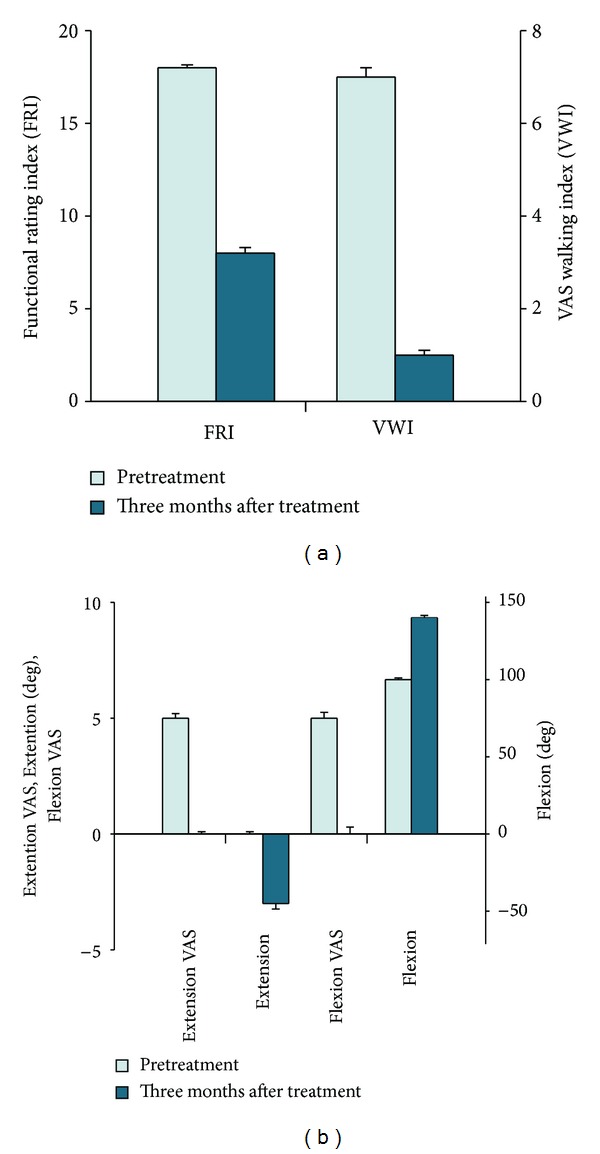
(a) Outcome of pain measurements. (b) Range of motion from the patient. VAS is visual analog scale and error bars indicate standard deviations (*n* = 3).

**Figure 2:**
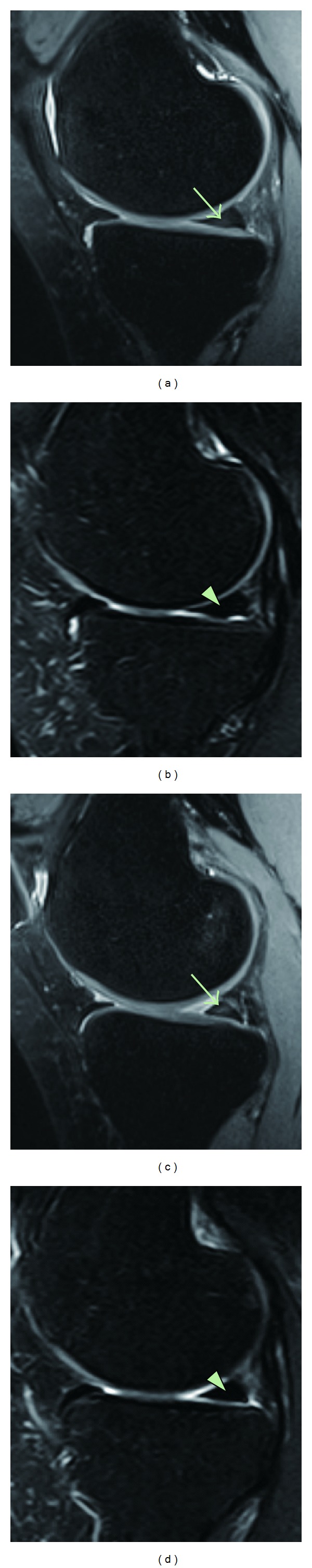
MRI sagittal sequential T2 views of the knee. Pretreatment MRI scans ((a) (sequential image: 5/20) and (c) (6/20)) show a tear (arrow) within the posterior horn of the medial meniscus. Posttreatment MRI scans at three months ((b) (5/20) and (d) (6/20)) indicate the healed meniscus (triangle) that has been repaired by ASCs mixture-based treatment.

**Figure 3:**
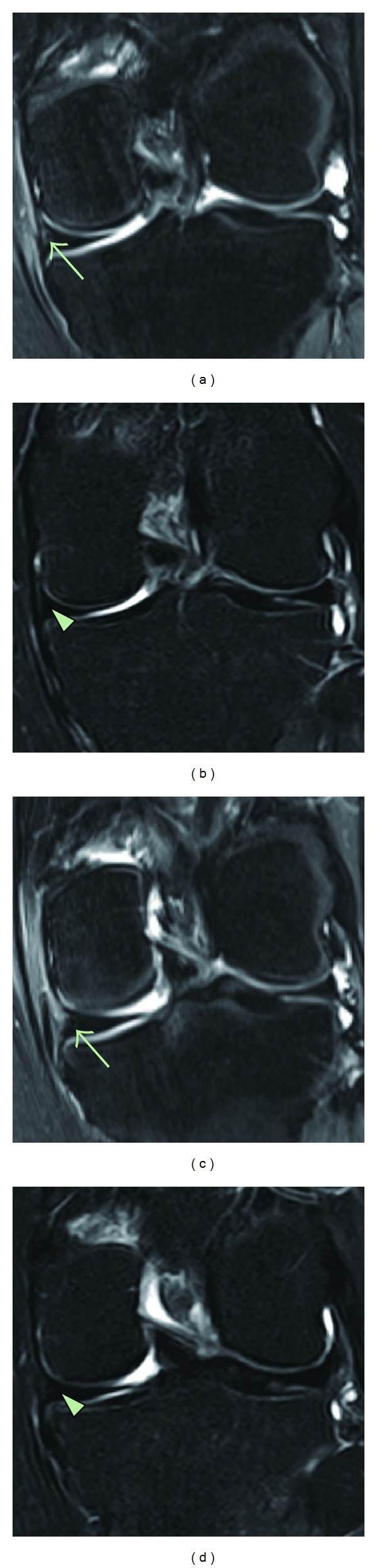
MRI coronal sequential T2 views of the knee. Pretreatment MRI scans ((a) (sequential image: 14/20) and (c) (15/20)) show a tear (arrow) within the posterior horn of the medial meniscus. Posttreatment MRI scans at three months ((b) (14/20) and (d) (15/20)) indicate the healed meniscus (triangle) that has been restored by ASCs mixture-based treatment.

A 32-year-old female has been experiencing left knee pain for the last two years prior to the office visit. The patient denied any history of significant trauma, except exercising on a treadmill. On the day of the initial evaluation, she reported moderate pain (visual analog scale (VAS) score of 5) on rest and increased pain when walking (VAS walking index (VWI) of 7; Figure 1(a)). She also complained of mild knee swelling. On physical examination, there were mild knee joint edema, minimally decreased range of motion, and mild tenderness with flexion. Apley and McMurray's tests were questionably negative, and there was no ligament laxity. To rule out bursitis, osteoarthritis, tendinitis, and muscle spasm, all of which could cause similar types of knee pain and must be carefully sought in this study, an MRI of the knee was performed. The MRI showed Grade II meniscal tear (Figures 2(a), 2(c), 3(a), and 3(c)). Owing to continuous pain (functional rating index (FRI): 18; Figure 1(a)), the patient initially tried treatments comprising nonsteroidal anti-inflammatory drugs, physical therapy, PRP, and hyaluronic acid. However, these therapies failed.

When such conservative treatment fails, an arthroscopic resection of the meniscus is necessary [1]. The major drawback of the meniscectomy is the early onset of osteoarthritis of the knee [83]. With the increasing recognition of the meniscus as an important structure of the knee, meniscal repair has become the preferred treatment of choice over meniscectomy [84].

From June 2009, autologous, noncultured ASCs can now be used as a source of MSCs in Korea [11]. Being aware of the fact that ASCs have been allowed by Korean Food and Drug Administration, and being wary of the possibility of early onset of osteoarthritis, the patient wanted to try autologous noncultured ASC mixture-based treatments for the repair of the damaged meniscus.

After obtaining autologous ASCs and preparing PRP as previously described [4, 5], the ASC mixture was percutaneously injected under the ultrasound guidance into the knee joint of the patient. Three months after the treatment, the patient's symptoms improved (Figures 1(a) and 1(b)) and repeated MRI showed almost complete disappearance of the torn meniscus (Figures 2(b), 2(d), 3(b), and 3(d)).

The patient was offered the sixth, twelfth, and eighteenth month postprocedure MRIs, but she refused to undergo postprocedure MRIs due to symptom improvement and financial reasons. Therefore, the longer-term (more than three months) followups were conducted based on telephone questionnaires. Until 18 months, her symptom improvement was persistent and she did not report any serious side effects (cancer or any complications). A recent report supports this result [85]. Because ASCs and PRP are autologous in nature, no rejection was expected and none occurred.

## 4. Discussion

A diagnostic MRI of the knee was performed on the patient before ASC mixture-based treatment. Consequently, posttreatment MRIs were performed to compare pre- and posttreatment images. The MRI T2 sequence was used for its ability to show bony anatomy. Due to slight differences in patient positioning and slight movement of the patient during the MRI procedures, there was some difficulty in capturing the exact and identical treatment location. However, the pre-and posttreatment MRI results can be compared with sequential views to compensate for any possible errors [12].

In the clinical result of ASC mixture application, significant MRI signal changes were apparent in the T2 views of the knee along injection sites (the medial Tibiofemoral joint and the medial inferior retropatellar joint). These significant signal changes can be interpreted as signs of persistent and restored meniscus. Due to reduction in the knee pain, the patient was reluctant to undergo a knee meniscal biopsy to determine the true nature of the cartilage-like tissue. Although the true nature of the restored tissue is unclear, the torn meniscus is believed to be restored, based on previous studies showing cartilage recovery using mesenchymal stem cells (MSCs) in patients with osteonecrosis, osteoarthritis, and chondromalacia patellae [9, 10]. The reduction in the knee pain and significant MRI signal changes support this notion.

With regard to the mechanism of the possible regenerative meniscal repair, there are few plausible possibilities: (i) direct differentiation of the stem cells (e.g., ASCs) [21–43]; (ii) trophic and paracrine effects of ASCs on the existing tissue (e.g., meniscal cartilage) [44–60]; (iii) effects of growth factors contained in PRP [61–66, 69, 70]; (iv) the ASCs' differentiation induced by low physiologic doses of dexamethasone [71–80]; or (v) combination of all of the above-mentioned possibilities. Based on previous reports [9–12] and this clinical result, it is evident that ASCs most likely play an important role in the repair of the torn meniscus. To our knowledge, this is the first report of a successful restoration of the torn meniscus by using the new ASC mixture-based regimen.

It has been estimated that approximately 400,000 ASCs are contained in 1 mL of adipose tissue [86]. Because 40 mL centrifuged adipose tissue was harvested, it is believed that approximately 16,000,000 stem cells were extracted and injected into the knee joint.

Although this clinical report was not a randomized and controlled trial, the key clinical feature of this clinical result is demonstrating the possible availability of potentially effective and noninvasive treatment in patients with continuous knee pain due to meniscus tear. In addition, although there was no placebo (ASC-free) group, the patient had received PRP and hyaluronic acid injections without ASCs. This treatment did not provide any clinical improvement. The patient experienced significant reduction of the knee pain only after percutaneous injection of ASC mixture.

## Figures and Tables

**Table 1:** Inclusion criteria.

Description	
(1) MRI evidence of meniscal tear	
(2) Orthopedic evaluation that determined that patient was a candidate for an arthroscopic meniscectomy	
(3) Either male or female	
(4) Under 60 years of age	
(5) An unwillingness to proceed with arthroscopic resection of the meniscus	
(6) The failure of conservative management	
(7) Ongoing pain	

**Table 2:** Exclusion criteria.

Description	
(1) Active inflammatory or connective tissue disease thought to affect the patient's pain (i.e., lupus, rheumatoid arthritis, fibromyalgia)	
(2) Active endocrine disorder that might affect the patient's pain (i.e., hypothyroidism, diabetes)	
(3) Active neurological disorder that might affect the patient's pain (i.e., peripheral neuropathy, multiple sclerosis)	
(4) Active cardiac disease	

**Table 3:** Outcome endpoints (obtained at three months after treatment).

Description	
(1) Pre- and posttreatment VAS (visual analog scale) walking index	
(2) Pre- and posttreatment functional rating index	
(3) Pre- and posttreatment range of motion	
(4) Pre- and posttreatment MRI (magnetic resonance imaging)	
